# Preoperative prediction of nodal status using clinical data and artificial intelligence derived mammogram features enabling abstention of sentinel lymph node biopsy in breast cancer

**DOI:** 10.3389/fonc.2024.1394448

**Published:** 2024-07-10

**Authors:** Cornelia Rejmer, Looket Dihge, Pär-Ola Bendahl, Daniel Förnvik, Magnus Dustler, Lisa Rydén

**Affiliations:** ^1^ Department of Clinical Sciences, Division of Surgery, Lund University, Lund, Sweden; ^2^ Department of Plastic and Reconstructive Surgery, Skåne University Hospital, Malmö, Sweden; ^3^ Department of Clinical Sciences, Division of Oncology, Lund University, Lund, Sweden; ^4^ Medical Radiation Physics, Department of Translational Medicine, Lund University, Malmö, Sweden; ^5^ Department of Hematology, Oncology and Radiations Physics, Skåne University Hospital, Lund, Sweden; ^6^ Diagnostic Radiology, Department of Translational Medicine, Lund University, Malmö, Sweden; ^7^ Department of Surgery, Skåne University Hospital, Malmö, Sweden; ^8^ Department of Clinical Medicine, Aarhus University, Aarhus, Denmark

**Keywords:** breast cancer, de-escalation, sentinel lymph node biopsy, artificial intelligence, mammography, prediction model, personalized medicine

## Abstract

**Introduction:**

Patients with clinically node-negative breast cancer have a negative sentinel lymph node status (pN0) in approximately 75% of cases and the necessity of routine surgical nodal staging by sentinel lymph node biopsy (SLNB) has been questioned. Previous prediction models for pN0 have included postoperative variables, thus defeating their purpose to spare patients non-beneficial axillary surgery. We aimed to develop a preoperative prediction model for pN0 and to evaluate the contribution of mammographic breast density and mammogram features derived by artificial intelligence for de-escalation of SLNB.

**Materials and methods:**

This retrospective cohort study included 755 women with primary breast cancer. Mammograms were analyzed by commercially available artificial intelligence and automated systems. The additional predictive value of features was evaluated using logistic regression models including preoperative clinical variables and radiological tumor size. The final model was internally validated using bootstrap and externally validated in a separate cohort. A nomogram for prediction of pN0 was developed. The correlation between pathological tumor size and the preoperative radiological tumor size was calculated.

**Results:**

Radiological tumor size was the strongest predictor of pN0 and included in a preoperative prediction model displaying an area under the curve of 0.68 (95% confidence interval: 0.63–0.72) in internal validation and 0.64 (95% confidence interval: 0.59–0.69) in external validation. Although the addition of mammographic features did not improve discrimination, the prediction model provided a 21% SLNB reduction rate when a false negative rate of 10% was accepted, reflecting the accepted false negative rate of SLNB.

**Conclusion:**

This study shows that the preoperatively available radiological tumor size might replace pathological tumor size as a key predictor in a preoperative prediction model for pN0. While the overall performance was not improved by mammographic features, one in five patients could be omitted from axillary surgery by applying the preoperative prediction model for nodal status. The nomogram visualizing the model could support preoperative patient-centered decision-making on the management of the axilla.

## 1 Introduction

Sentinel lymph node biopsy (SLNB) is the recommended surgical axillary staging method in patients with clinically node-negative breast cancer, although approximately 75–80% have non-malignant axillary lymph nodes in the definitive pathology report (1–4). Consequently, patients with negative sentinel lymph node status (pN0) do not benefit from SLNB. The American College of Surgeons Oncology Group Z0011(ACOSOG Z0011) study questioned the necessity of axillary lymph node dissection and showed that abstaining from ALND in patients with T1-T2 clinically node negative primary breast cancer with 1-2 sentinel lymph nodes containing metastases was non-inferior to ALND regarding overall survival. This raised the question of the necessity of SLNB. The randomized Sentinel Node vs Observation After Axillary Ultra-Sound (SOUND) trial recently showed that abstaining SLNB in patients with T1 tumors having breast-conserving surgery was non-inferior to SLNB regarding distance-free survival at five years (5). However, implementation of the findings from the SOUND trial is not applicable to all breast cancer patients. The ASCO guidelines already recommended abstaining from SLNB in 2021 for patients ≥ 70 years with a luminal subtype undergoing breast-conserving surgery and the recommended adjuvant endocrine therapy (2). There is an increasing awareness of the importance of the long-term effects of surgery on patient’s function and well-being. The Intergroup Sentinel Mamma study (INSEMA) evaluating invasive disease-free survival and morbidity after breast-conserving surgery with or without SLNB reported that morbidity was lower in the group without SLNB than in the group with SLNB at one, three, and 18 months postoperative (3). This warrants strategies for implementation of de-escalation of axillary surgery and methods for preoperative identification of patients for whom SLNB can be safely omitted.

Several clinicopathological models for prediction of axillary lymph node (ALN) and sentinel lymph node (SLN) status have been developed during the past decade (6–9). In 2019, Dihge et al. (10) developed an artificial neural network (ANN) model to predict pN0. The selected variables in the model are well known predictors and most were previously included in Bevilacqua et al.’s prediction model (7, 10). However, previous prediction models were developed using postoperatively available variables, defeating the purpose of a patient-centric preoperative decision tool for safe omission of SLNB. A commonly used key predictor for pN0, pathological tumor size, is a postoperative measure that could be replaced by radiological tumor size. Studies have indicated that primarily mammographic tumor size is similar to the postoperatively available pathologic tumor size, while other imaging modalities often over- or underestimate the tumor size (6, 7, 11). To our knowledge, a comparison of pathological tumor size and radiological tumor size has not previously been described in the setting of ALN status prediction.

Prediction models for ALN status using mammograms have been presented using presence of microcalcifications, breast density, and radiomic signatures, exclusively or in combination with clinicopathological variables, most of which were postoperatively obtained (12–15). In addition, several studies have investigated using other breast imaging modalities for nodal prediction, including ultrasound and contrast-enhanced mammography (16–21). To our knowledge, no prediction model for pN0 currently incorporates commercially available AI cancer detection features from mammograms and exclusively preoperatively available clinicopathological variables.

Thus, we aimed to evaluate non-operative nodal staging and the possibility to omit axillary surgery by developing a prediction model for pN0 using only preoperatively available data. The additional predictive value of mammographic variables extracted by a commercially available AI cancer detection system and an automated breast density assessment system in patients with clinically node-negative primary breast cancer is explored. A nomogram is developed as a preoperative decision-tool to enable a patient-centered approach to SLNB. Additionally, we aimed to evaluate radiological tumor size as a preoperative alternative to the postoperative pathological tumor size as a predictor in a preoperative prediction model for pN0.

## 2 Materials and methods

### 2.1 Study population

A total of 770 women diagnosed with primary breast cancer between January 2009 and December 2012 were prospectively included in a registry at the Department of Pathology at the Skåne University Hospital (Lund, Sweden). Patients with clinically node negative primary breast cancer undergoing primary breast surgery and SLNB were included as previously described by Dihge et al. (6, 10). Clinically node negative was defined as no palpable mass in the physical examination. All patients underwent SLNB as axillary staging, and if needed, axillary lymph node dissection. Another cohort including 586 patients from Skåne University Hospital (Malmö, Sweden in 2020) and Helsingborg Regional Hospital (Helsingborg, Sweden in 2019–2020) was used for external validation (22).

### 2.2 Clinicopathological data

Patient and tumor information was collected from patients’ electronic files and a pathology database, as described by Dihge et al. (6, 10) and Skarping et al. (22). The histological type was divided into two groups after variable selection: the first group included no special type and lobular, and the second group included other and mixed types (7). Estrogen receptor (ER) status, progesterone receptor (PR) status, HER2 status, and Ki67 percentage were analyzed and categorized according to guidelines (23, 24). Mode of tumor detection was divided into symptomatic presentation and by the national mammography screening program. Tumor localization was defined by location in the four quadrants and central, and after statistical variable selection, categorized as upper inner quadrant vs. other locations (7).

SLN status was categorized as negative or positive (pN0 or pN+). pN0 was defined as breast cancer without lymph node metastasis or with isolated tumor cells. pN+ was defined as ≥1 SLN with micrometastasis or macrometastasis, defined as >200 cells and/or cluster size of 0.2 – 2 mm, and cluster size >2 mm, respectively (25).

### 2.3 Mammographic images and image analysis systems

All available mammographic images from screening and diagnostic imaging were included in the analyses. A modified Breast Imaging Reporting and Data System malignancy score (1–5) was used for mammographic and ultrasound images, as they are part of the clinical routine work-up. For this study mammographic malignancy score (1–5), ultrasound malignancy score (1–5), the largest specified radiologic tumor size in mm, and laterality were collected from the Picture Archiving and Communication System (PACS). In cases of missing mammographic tumor size, size from ultrasound was used since both modalities are preoperative and included in the initial clinical work-up. Mammography has been shown to have a high accuracy when compared to the surgical specimen, while ultrasound tends to underestimate the tumor size (26).

Transpara (version 1.7.0, Screenpoint Medical, Nijmegen, the Netherlands), a breast cancer detection tool uses deep learning algorithms to detect suspicious soft tissue lesions and microcalcifications (calc) that may indicate breast cancer. Each suspicious region is assigned a score between 1 and 100. When used for screening mammography, Transpara sorts cases into ten different risk categories (1-10) based on the regional suspicion scores. It is calibrated to sort roughly equal numbers of cancers into each category, with a goal of 90%+ of cancers in category 10 (27). Several retrospective studies (28–31) have shown Transpara to be effective in increasing cancer detection and reducing workload in screening, and the prospective Mammography Screening with Artificial Intelligence (MASAI) trial has demonstrated it to be effective in a clinical screening setting (32). Additionally, Transpara has been reported to predict stage II breast cancer years before diagnosis, indicating detection of properties beyond the cancer diagnosis (33). For this study, the highest calc cluster score and soft tissue lesion score were extracted from Transpara. The scores were included in the set of candidate predictors as continuous variables (0–100) and dichotomized as presence of finding (0 vs 1–100). All available mammograms were included and were manually cross-checked for laterality and correct tumor localization in Transpara before data extraction.

LIBRA (version 1.0.5) is an automated breast density estimation algorithm, which analyzes images based on gray-level values and segments them into dense and non-dense areas, developed at the University of Pennsylvania (17). Gastounioti et al. (34) and Keller et al. (35), among others, have validated LIBRA as a breast density measurement system. In this study, dense area [cm2] and density [%] were extracted from LIBRA. Craniocaudal and mediolateral oblique projections were available for all patients and therefore included in the analyses. The mean values from LIBRA of the projections on the ipsilateral side were used as the contralateral side was not available for all patients. Moreover, several studies revealed an association between breast density and breast cancer as well as with ALN status (33–38).

### 2.4 Statistical analysis

Descriptive analyses were performed to explore the associations between clinical, pathological, and radiological variables, and SLN status using the Mann–Whitney U test and Chi-square test for continuous and categorical variables, respectively. Pathological and mammographic tumor size were compared using Pearson correlation and Bland-Altman analysis. Univariable logistic regression was used for radiological variables to predict pN0. The top ten variables included in the model published by Dihge et al. (10) were used as a framework and included in a multivariable logistic regression (MLR) analysis. In the published article, an ANN model was developed with cross-validation and compared with an MLR model. The performance of the simpler MLR model was found to be marginally inferior. Therefore, we proceeded with the MLR model (area under the receiver operating characteristics curve (AUC) 0.73) in the present study, hence referred to as the postoperative framework model (10). In this study, vascular invasion and multifocality were excluded due to clinical unavailability or poor preoperative quality. The postoperative variables, Ki67 and pathological tumor size, were exchanged for the preoperatively available variables, histological grade and radiological tumor size. Stepwise backward elimination MLR with a p-value threshold for removal of 0.157 was performed to obtain a clinical preoperative model. Radiological variables were evaluated as additional candidates to improve the clinical preoperative model, using stepwise backward elimination. The model stability was assessed by performing the model selection procedure in 1000 bootstrap samples as well as by five-fold cross-validation repeated ten times. Prediction models were compared using AUC and the Akaike information criterion (AIC). The SLNB reduction rate was calculated with a cut-off based on a 10% false negative rate (FNR), reflecting the clinically accepted FNR of SLNB (39, 40). In addition, SLNB reduction rate was calculated with a 20% FNR for comparison. Point estimates for the clinical preoperative prediction model were illustrated by a nomogram. The proposed prediction model was externally validated, temporally and geographically, in a separate cohort. The calibration in the validation cohort was evaluated using the Hosmer-Lemeshow approach. Briefly, the predicted probabilities of pN0 were plotted against the mean predicted probability of pN0 according to the model. Perfect calibration will hence respond to 10 plot symbols on a line with a 45-degree slope.

P-values were not corrected for multiple comparisons due to the explorative nature of the study. All p-values are two-sided and interpreted as level of evidence against the null hypothesis without reference to a cut-off for significance. Stata (StataCorp. 2021. Stata Statistical Software: Release 17. College Station, TX; StataCorp LLC) was used for all statistical analyses.

## 3 Results

**Figure 1:**
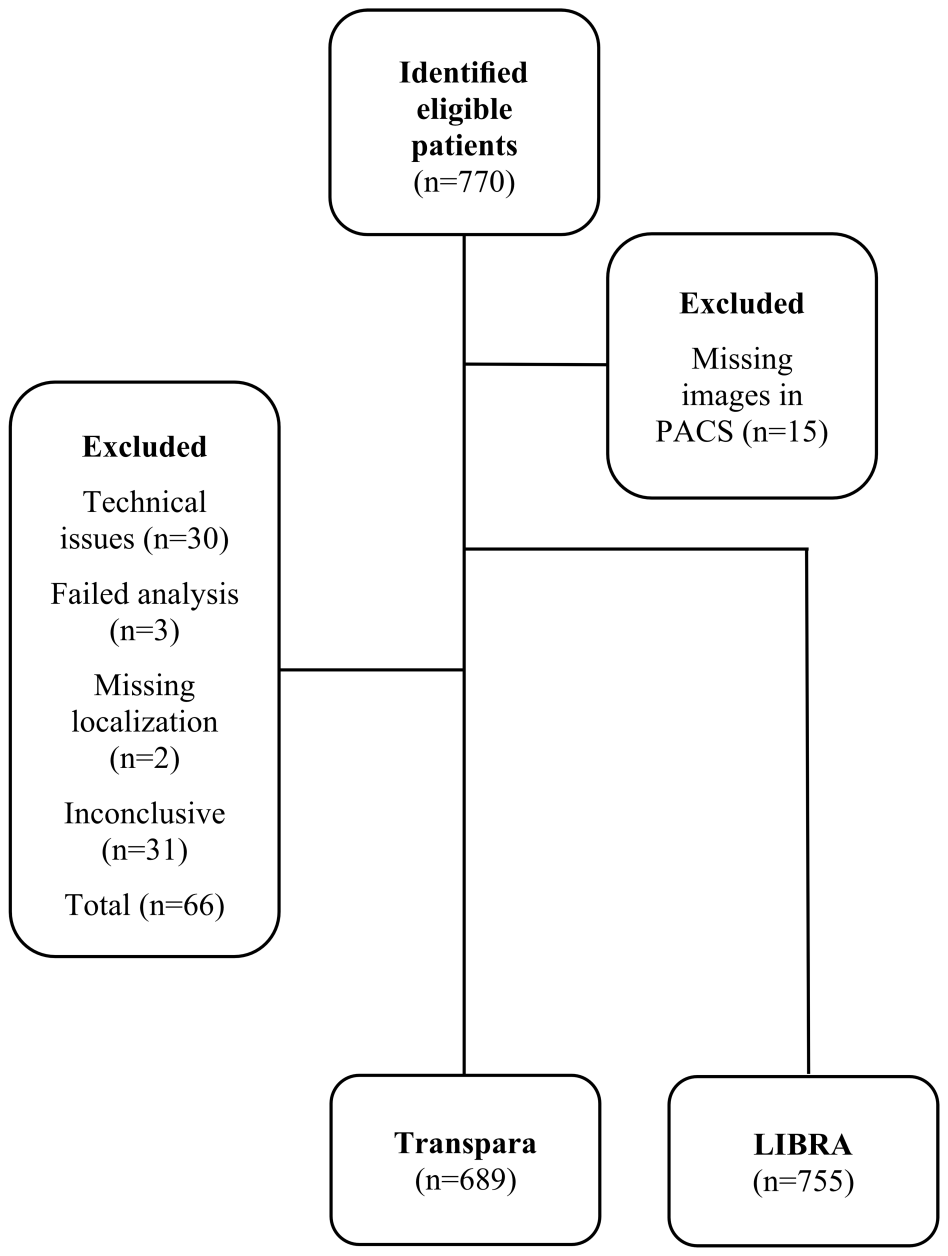
Flow chart of patient inclusion. At inclusion there were missing images (n=15) in PACS, additional images (n=30) were excluded due to technical issues. Abbreviations: Laboratory for Individualized Breast Radiodensity Assessment (LIBRA); Picture Archiving and Communication System (PACS).

Mammograms were identified in 755 of the 770 patients included in the study. All patients and images were included in the LIBRA subgroup. Transpara failed to analyze mammograms from three patients and 30 patients were excluded due to technical issues in PACS. Inconclusive cases, according to radiologists, and cases without a clear indication of tumor location in PACS were excluded due to the inability to cross-check the AI findings. The inclusion and exclusion of patients with annotated mammograms are presented in **Figure 1**. The AI assessment of tumor localization on mammograms was correct in 96.1% of the cases.

The patient, tumor, and radiological characteristics of the primary cohort are presented in **Table 1** and the external validation cohort in **Supplementary Table 1**. Patient and tumor characteristics were similar in the two cohorts, apart from the prevalence of pN0. In the primary cohort, 35% were pN+, while only 26% were pN+ in the external cohort. The patient and tumor characteristics that showed the strongest evidence for association with pN0 were pathological tumor size (p <0.001), mode of tumor detection (p <0.001), multifocality (p <0.001), vascular invasion (p <0.001), Ki67 (p <0.001), histological grade (p=0.007), age (p=0.027), and histological type (p=0.046). The radiological variables strongest associated with pN0 were radiological tumor size (p <0.001), and the highest soft tissue lesion score (p <0.001).

**Table 1:** Patient, tumor, and radiological variables stratified by sentinel lymph node status.

	All (n=770)	pN0 (n=501)	pN+ (n=269)	*P*
Age, years (continuous)* ^**c**^	64.7 (24.2–91.9)	65.9 (32.6–91.5)	64.2 (24.2–91.9)	0.027^a^
Pathological tumor size, mm (continuous)* ^**d**^	15 (0.5–90)	13 (0.5–55)	18 (0.9–90)	<0.001^a^
Missing	1	1	0	
Mode of tumor detection**^c^				<0.001
Symptomatic	321	184 (37)	137 (51)	
Screening	449	317 (63)	132 (49)	
Multifocality**^d^				<0.001^b^
Yes	186	96 (19)	90 (33)	
No	584	405 (81)	179 (67)	
Tumor localization** ^**c**^				0.108^b^
Central	22	14 (3)	8 (3)	
Upper inner	105	76 (15)	29 (11)	
Lower inner	46	32 (6)	14 (5)	
Upper outer	253	160 (32)	93 (35)	
Lower outer	78	41 (8)	37 (14)	
Overlapping	266	178 (36)	88 (33)	
Histological type** ^**c**^				0.046^b^
NST and lobular	713	457 (91)	256 (95)	
Other or mixed	57	44 (9)	13 (5)	
Histological grade** ^**c**^				0.007^b^
I	186	137 (28)	49 (18)	
II	350	224 (45)	126 (47)	
III	226	133 (27)	93 (35)	
Missing	8	7	1	
Vascular invasion** ^**d**^				<0.001^b^
Yes	91	27 (6)	64 (32)	
No	526	390 (94)	136 (68)	
Missing	153	84	69	
ER status** ^**c**^				0.056^b^
Negative	69	53 (11)	16 (6)	
Positive	699	446 (89)	253 (94)	
Missing	2	0	2	
PR status** ^**c**^				0.077^b^
Negative	122	89 (18)	33 (12)	
Positive	646	410 (82)	236 (88)	
Missing	2	0	2	
HER2 status** ^**c**^				0.606^b^
Negative	624	411 (89)	213 (87)	
Positive	84	51 (11)	33 (13)	
Missing	62	39	23	
Ki67 (continuous)* ^**c**^	15 (0–94)	14 (0–94)	17 (1–81)	<0.001^a^
Missing	43	15	28	
Radiological tumor size, mm (continuous)* ^**c**^	15 (4–78)	13 (4–78)	17 (5–57)	<0.001^a^
Missing	179	119	60	
Highest calc cluster score (continuous)* ^**c**^	0 (0–98)	0 (0–98)	0 (0–97)	0.445^a^
Missing	81	44	37	
Calc cluster (binary)** ^**c**^				0.976^b^
Present	243	161 (35)	82 (35)	
Absent	446	296 (65)	150 (65)	
Missing	81	44	37	
Highest soft tissue lesion score (continuous)* ^**c**^	91 (0–97)	90 (0–97)	92.5 (0–97)	<0.001^a^
Missing	81	44	37	
Soft tissue lesion (binary)** ^**c**^				0.345^b^
Present	572	375 (82)	197 (85)	
Absent	117	82 (18)	35 (15)	
Missing	81	44	37	
Breast density, %* ^c^	16.8 (1.7–99.8)	16.1 (1.7–99.8)	18.7 (2.0–99.7)	0.215^a^
Missing	22	10	12	
Breast dense area, cm^2^* ^**c**^	22.9 (1.64–208.0)	22.2 (1.6–208.0)	23.4 (3.8–197.9)	0.529^a^
Missing	22	10	12	
Mammography malignancy score** ^**c**^				0.386^b^
1	26	16 (4)	10 (4)	
2	8	7 (2)	1 (0)	
3	59	41 (9)	18 (8)	
4	205	143 (32)	62 (28)	
5	369	236 (53)	133 (59)	
Missing	103	58	45	
Ultrasound malignancy score** ^**c**^				0.100^b^
1	35	28 (6)	7 (3)	
2	8	6 (1)	2 (1)	
3	32	22 (5)	10 (5)	
4	132	96 (22)	36 (16)	
5	453	286 (65)	167 (75)	
Missing	110	63	47	

Negative sentinel lymph node status (pN0), positive sentinel lymph node status (pN+), no special type (NST), estrogen receptor (ER), progesterone receptor (PR).

*Median (range).

**Number (%).

a Mann–Whitney U test.

b Chi-square test.

c Preoperatively available.

d Postoperatively available.

**Figure 2:**
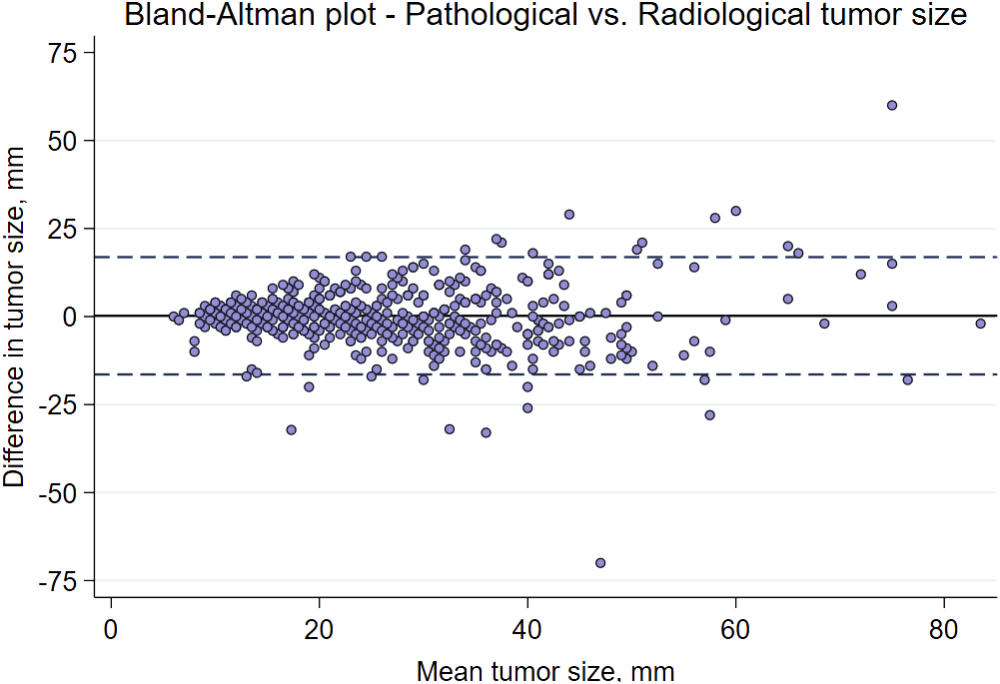
A Bland-Altman plot illustrating the difference in mean values between the preoperative radiological tumor size and the pathological tumor size from the surgical specimen.

A comparison of tumor size by pathological and mammographic assessment is presented in **Supplementary Table 2**. The agreement between tumor size variables was also evaluated in a Bland-Altman plot of differences vs. average (**Figure 2**). The mean pathological and radiological tumor sizes were 16.7 and 17.1 mm, respectively, and the Pearson correlation coefficient was 0.62.

The univariable logistic regression analyses of pN0 are presented in **Supplementary Table 3** and AUCs for radiological variables in **Supplementary Table 4**. Radiological tumor size (odds ratio (OR) 0.97 per mm, 95% confidence interval (CI) 0.95–0.98, p <0.001) had the strongest evidence of association with pN0 in univariable analyses.

**Figure 3:**
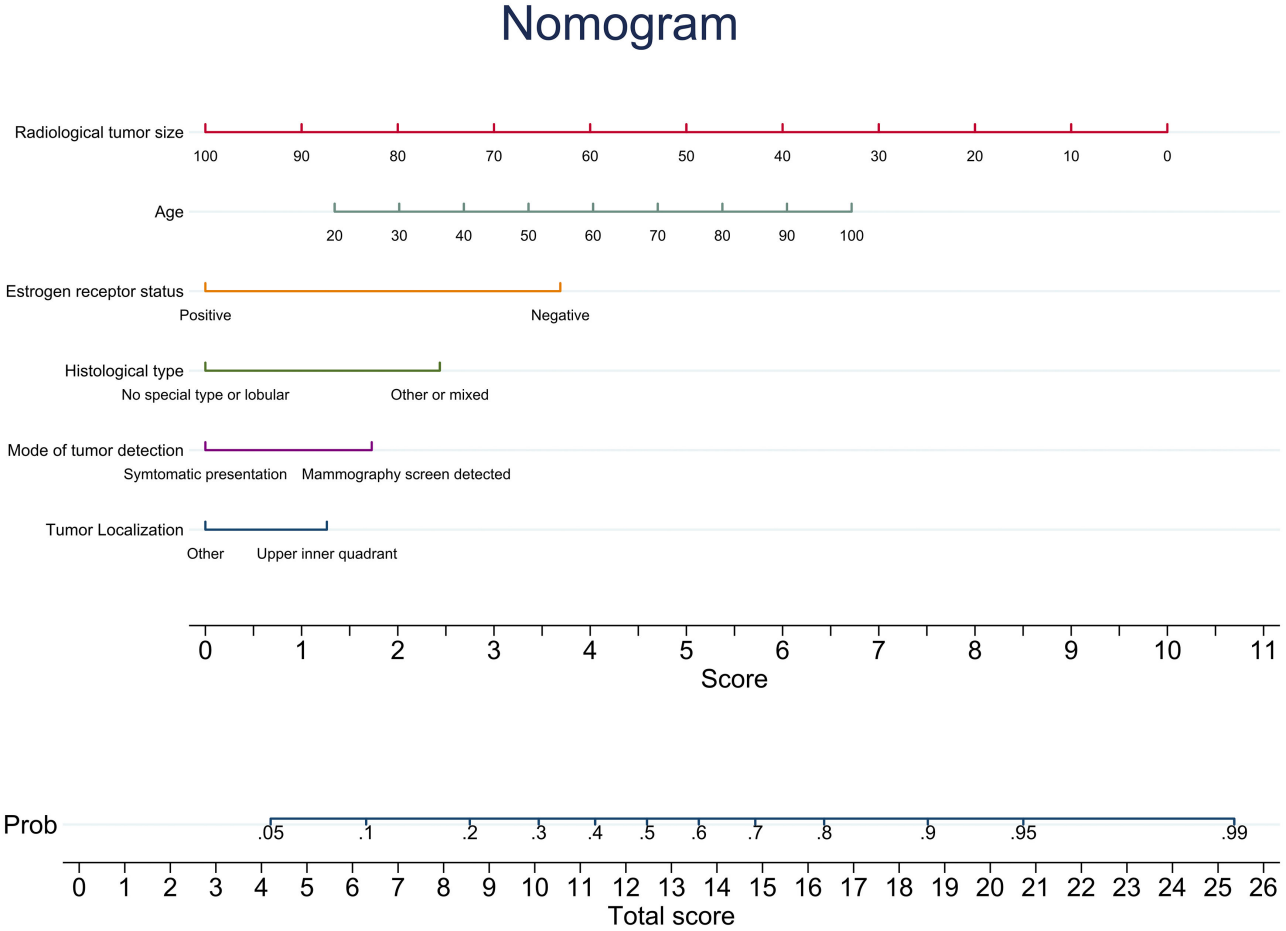
A nomogram illustrating the point estimates of the included variables’ coefficients for the clinical preoperative prediction model for prediction of negative sentinel lymph node status.

The MLR resulted in a clinical preoperative model including radiological tumor size, ER status, age, mode of detection, histological type, and tumor localization (upper inner quadrant vs. other), with an AUC of 0.68 (95% CI: 0.63–0.72) (**Table 2**). A nomogram visualizing the point estimates for the clinical preoperative model was developed (**Figure 3**). The remaining radiological variables added to this model using the same method, resulted in a combined preoperative model including radiological tumor size, ER status, age, mode of detection, histological type, tumor localization, highest soft tissue lesion score (continuous), and soft tissue lesion (binary) with an AUC of 0.68 (95% CI: 0.63–0.72) (**Table 2**). The corresponding AUC for the postoperative framework model was 0.76 (0.71–0.80). Each model’s AIC is presented in **Supplementary Table 5**. The candidate variable selection procedure was evaluated in 1000 bootstrap samples as well as by cross-validation. Radiological tumor size was selected in 96.5% of the bootstrap analyses (**Supplementary Table 6**) and in 100% of the cross-validation analyses. The clinical preoperative prediction model was externally validated with an AUC of 0.64 (95% CI: 0.59–0.69). A Hosmer-Lemeshow calibration plot is presented in **Supplementary Figure 1**. Applying the clinical preoperative prediction model to assign pN0 resulted in a possible SLNB reduction rate of 21% or 34% (**Table 3**), corresponding to a cut-off that accepts a 10% or 20% FNR, respectively.

**Table 2:** The clinical and combined preoperative prediction models for pN0. Backward variable selection with threshold p≥0.157 for removal.

	Clinical preoperative prediction model		Combined preoperative prediction model	
	OR (95% CI)	*P*	OR (95% CI)	*P*
Radiological tumor size, mm (continuous)*	0.965 (0.947–0.983)	<0.001	0.977 (0.964–0.990)	<0.001
**Age, years (continuous)**	1.024 (1.008–1.040)	0.003	1.018 (1.006–1.029)	0.002
**Mode of tumor detection**		0.001		0.001
Symptomatic	1 (reference)		1 (reference)	
Screening	1.847 (1.267–2.693)		1.565 (1.198–2.045)	
**Histological type**		0.025		0.035
Other or mixed	1 (reference)		1 (reference)	
NST or lobular	0.419 (0.196–0.899)		0.565 (0.332–0.961)	
ER status*		0.001		0.001
Negative	1 (reference)		1 (reference)	
Positive	0.269 (0.123–0.587)		0.403 (0.234–0.694)	
**Tumor localization**		0.100		0.063
Other	1 (reference)		1 (reference)	
Upper inner quadrant	1.553 (0.919–2.624)		1.417 (0.981–2.048)	
**Highest soft tissue lesion score (continuous)**			0.984 (0.968–1.001)	0.076
**Soft tissue lesion (binary)**				0.087
Absence			1 (reference)	
Presence			4.051 (0.814–20.15)	
**Constant**	3.724		1.862	

Negative sentinel lymph node status (pN0), odds ratio (OR), confidence interval (CI), no special type (NST), estrogen receptor (ER).

**Table 3:** SLNB reduction rate using the clinical preoperative prediction model to assign sentinel lymph node status.

FNR 10%				
	TP	TN	FP	FN
No.	189	105	276	20
SLNB reduction rate	(TN + FN)/(TP + TN + FP + FN) = 21%			
FNR 20%				
	TP	TN	FP	FN
No.	168	157	224	41
SLNB reduction rate	(TN + FN)/(TP + TN + FP + FN) = 34%			

Sentinel lymph node biopsy (SLNB), false negative rate (FNR), true positive (TP), true negative (TN), false positive (FP) and false negative (FN).

## 4 Discussion

In this study, a truly preoperative prediction model for pN0 in primary breast cancer was developed combining radiological and preoperatively available routine clinicopathological variables. The inclusion criteria were not restricted by age, tumor size, or type of surgery. This supports that the model can be used on a case-by-case basis evaluation of pN0 outside the ASCO guidelines on abstaining SLNB in older patients with ER+/HER2- tumors (2). Radiological tumor size was the strongest preoperative predictor of pN0 reflected by its low p-value and high selection rate (≈100%) in bootstrap analyses and cross-validation. This indicates that mammographic tumor size could replace pathological tumor size in preoperative models. Moreover, it was strongly associated with pathological tumor size. The soft tissue lesion score was associated with pN0 in univariable analyses, supporting the hypothesis that mammographic features could aid in preoperatively identifying these patients. However, although associated with the outcome, addition of radiological variables to the clinical preoperative model did not improve discrimination. The clinical and combined preoperative model had AUCs of 0.68 (95% CI: 0.63–0.72), indicating that the addition of radiological variables did not improve the overall performance of the model. External validation of the clinical preoperative prediction model resulted in an AUC of 0.64 (95% CI: 0.59–0.69). The Hosmer-Lemeshow calibration plot demonstrated that the prediction model underestimates the probability of node negativity, although the estimates follow the 45-degree line. A likely explanation is the difference in pN+ prevalence between the cohorts. Nevertheless, the clinical preoperative prediction model could putatively support the omission of SLNB in 21% of patients, if a 10% FNR is accepted, reflecting the accepted FNR of the SLNB procedure and support the implementation of the ASCO guidelines and the results of the SOUND trial (2, 5). The reduction rate is directly dependent on the accepted FNR. The FNR reflecting SLNB could be considered conservative, and accepting a higher FNR might be acceptable in clinical practice. Applying a 20% FNR resulted in a 34% SLNB reduction rate. An alternative to a fixed cut-point, enabling a more patient-centered care, would be to allow different cut-points to be discussed and decided with the patient, on a case-by-case basis.

Pathological tumor size is a strong predictor of SLN status and often included in published prediction models, although it is assessed on the postoperative surgical specimen (10, 12, 13). In accordance with previous research, pathologic and radiologic measurements of tumor size were strongly correlated. The correlation coefficient was 0.62, in the present study, which can be compared to the correlation between pathologic tumor size and radiologic tumor size measured by mammography or ultrasound, depending on histological subtypes, in a study by Gruber et al. (11). This indicates that radiologic tumor size could be used as an alternative measure for pathological tumor size. Mammography has also been shown to estimate the tumor size more accurately than ultrasound, which underestimates the size with a varying degree depending on the histological tumor type (11). In this study radiologic tumor size was strongly associated with pN0 indicating that mammographic tumor size could replace pathological tumor size as a predictor of pN0. However, there may be subgroups, such as patients with dense breasts, in which radiological tumor size needs further evaluation.

The clinical preoperative and combined preoperative models (AUC 0.68) had lower AUCs than the postoperative framework model (AUC 0.76). This was expected as strong predictors, determined on the surgical specimen, were excluded from the model to make it clinically useful in a preoperative setting. The AIC of the postoperative framework prediction model was lower than those of the clinical preoperative and combined preoperative models, which was expected considering the superior discriminative capacity. Additionally, the AUC of the clinical preoperative prediction model was slightly lower in the external validation cohort, which was expected. The difference may be due to differences in prevalence of pN0 in the cohorts, where the external validation cohort had a higher prevalence. Additional analyses adjusting for the prevalence (data not shown) showed an AUC similar to the AUC of the internal validation. A prediction model for heavy nodal burden by Meteroja et al. (41) included prevalence of the outcome as a variable in the model to adjust for differences between populations. This is, however, not applicable in the present study due to the single center approach.

When elaborating on the radiological variables used in this study, it is important to note that Transpara was not intended to be used as a tool for SLN status prediction, although this study indicates its potential predictive value. Additional development of Transpara features in this direction may improve its predictive ability for pN0. However, the potential clinical use and definitions of medicolegal regulations regarding this type of diagnostic tools are debated and yet to be determined before clinical implementation (27, 42). Other forms of AI such as feature extraction from other imaging modalities, such as ultrasound, magnetic resonance imaging, and computed tomography, as well as machine learning methods have been evaluated for prediction of ALN status in several studies receiving high AUCs (16, 18–20). However, to our knowledge, none are yet available for implementation in clinical practice. Implementation of image analysis software in clinical practice could have other applications apart from screening and should therefore be evaluated for other possibilities (43). The implementation of a prediction model in clinical practice would entail additional costs, whereas omission of SLNB would likely reduce costs associated with surgery as previously described for the ANN model proposed by Dihge et al. (10, 44). Striving for de-escalation in cancer care, omission of SLNB could also improve the quality of life and reduce postoperative morbidity, as reported in the INSEMA trial (3). The ASCO guidelines on management of the axilla in early-stage breast cancer stated that patients should be evaluated on a case-by-case basis to ensure oncological safety (2). Therefore, a truly preoperative prediction model for pN0 based on routine information on the individual patients would be of high clinical relevance and act as a foundation for patient-centered decision making. The presented nomogram could be an easy-to-use decision tool to support the preoperative multidisciplinary decision-making to omit SLNB for one in five patients with predicted pN0 status, consequently reducing the succeeding complications. Considering the preoperative nature of the proposed model, it could be considered an improvement compared to the previously published prediction model, regardless of the inferior discriminative capacity. However, the proposed model was developed and validated in retrospective cohorts, thus requiring additional research to ultimately benefit patients. In order to enable implementation of the clinical preoperative prediction model, the model should be validated in prospective studies.

A limitation of previous clinical prediction models is that key variables can only be obtained postoperatively (6, 7, 10). In other studies, this problem was circumvented by including radiological variables from different imaging modalities exclusively or in addition to clinicopathological variables. Liu et al. proposed an exclusively radiological ANN model using contrast-enhanced computed tomography (16). However, this approach is only feasible for clinical implementation for patients with breast cancer who undergo contrast-enhanced computed tomography during the initial routine work-up, an argument which also applies to models that include magnetic resonance imaging (18, 19). Given the wide implementation of mammography as a cornerstone in the clinical work-up for suspicious breast cancer including screening programs, mammographic images are available for all patients and can be used for preoperative diagnostics. Cen et al. proposed a model that included postoperative clinicopathological variables and microcalcification density on mammographic images, resulting in a model with an AUC of 0.70 (12). In the study, microcalcification density >20 cm^2^ was associated with a positive ALN status. This was not observed in the present study, which might be a result of differing measuring techniques. Yang et al. (14) created a prediction model for ALN status (n=147) using a radiomic signature on mammography with an AUC of 0.88 in the validation cohort, but no independent validation has hitherto been performed. Studies have shown a positive association between breast density and malignant axillary lymph nodes when measured by radiologists and automated methods (36, 38), but when evaluated in a prediction model for pN0 including multifocality, pathological tumor size, histological type, Ki67 and histological grade, Hack et al. (13) found no additional predictive value, which is in line with the present study. In this study, images from the ipsilateral side were included in the LIBRA analysis. This was decided as mammographic images on the contralateral side were not available for all patients. The variation in tumor size (0.5 – 90 mm) can be assumed to have affected the results of the breast density variable in descriptive and univariable logistic regression as well as the performance of the variable in the MLR to some extent. However, the results are in accordance with previous results (13). LIBRA, which is a fully automated assessment tool that analyzes processed images, has been validated for breast density measurements on mammographic images by Gastounioti et al. (34) and Keller et al. (17), among others. The discrepancy between previous reports on the association between breast density and ALN status (13, 36, 38) could be due to differences between methods as revealed by Keller et al. (35).

A strength of this study is the relatively large cohort of 770 patients and that the inclusion criteria were not restricted by age, tumor size or type of surgery. All eligible cases during a four-year period were consecutively included, and the cohort should therefore be representative of the breast cancer population at Skåne University Hospital in Lund during the time period. Another strength is the assessment of Transpara’s accuracy through cross-checking for the correct tumor location. Regardless of the cross-checking, all cases were included to resemble a clinical setting. The pN0 nomogram presents a graphical easy-to-interpret visualization of the included predictors and the relative importance of each independent variable is visible at a glance. There are several limitations to this study, such as the low prevalence of pN0 compared to recent cohorts. This could be due to the fact that breast cancers are discovered at an earlier stage now than in past decades. Another limitation is the exclusion of 30 cases due to technical issues in the Transpara sub-cohort, a cause of which could not be identified despite repeated contact with the technical support at the hospital and PACS provider. The authors believe that the reason may be a technical issue with the PACS provider during the archiving process. However, the missing images represent less than 4% of the cohort and the authors have found no reason to believe that the missingness is systematic. The impact of the technical issues on the model development should therefore be minimal. Additionally, Transpara failed to analyze the images of three patients (<0.4%), with unspecified errors. Furthermore, radiological tumor size was missing in 23% of cases, likely due to the fact that the radiological tumor size measurement was not always provided at the time of inclusion of patients in the present study. Thus, the performance of the radiological variables could be biased owing to the missing data. The inclusion of sonographic tumor size in cases where mammographic size was not available has likely decreased the correlation between pathological and radiological tumor size as ultrasound underestimates the size. The correlation can be expected to be higher in a cohort using only mammographic tumor size, increasing the performance of the model. Considering the high correlation presented in this study and the performance of the radiological tumor size in all analyses, the inclusion of sonographic data should not have affected the results. Another limitation is the inclusion of only ipsilateral images in the LIRBA analysis, however, the effect on the overall conclusions can be presumed to be limited. Additionally, the prediction model on which this study is based is an ANN model that has the potential to capture non-linear associations and interactions, whereas the MLR model used in this study captures only linear effects on the log odds scale. This is a limitation and a strength, as the risk of overfitting is lower with less complex models such as MLR than with complex models. Moreover, to minimize the number of variables compared with the number of pN0 patients in the cohort, no interaction variables were included.

### 4.1 Conclusion

Radiological tumor size was strongly predictive of SLN status, thus supporting the hypothesis that radiological tumor size could replace pathological tumor size as a predictor of pN0. Additionally, although they did not improve the clinical preoperative prediction model, mammographic features might have nodal predictive capabilities. The presented clinical preoperative prediction model is visualized by a nomogram that could support the preoperative multidisciplinary decision-making to omit SLNB on a case-by-case basis for one in five patients with clinically node negative primary breast cancer with predicted pN0 status.

## Data availability statement

The datasets presented in this article are not readily available because of sensitive information according to current data legislation but are available from the corresponding author upon reasonable request. Requests to access the datasets should be directed to cornelia.rejmer@med.lu.se.

## Ethics statement

The study involving humans were approved by The Regional Ethical Review Board of Lund, Sweden, and KVB Samråd, Region Skåne. The study were conducted in accordance with the local legislation and institutional requirements. Written informed consent for participation was not required from the participants or the participants’ legal guardians/next of kin in accordance with the national legislation and institutional requirements.

## Author contributions

CR: Data curation, Formal analysis, Methodology, Visualization, Writing – original draft, Writing – review & editing, Validation. LD: Data curation, Writing – review & editing, Investigation. P-OB: Writing – review & editing, Conceptualization, Formal analysis, Methodology, Validation. DF: Writing – review & editing, Investigation, Resources. MD: Conceptualization, Resources, Writing – review & editing. LR: Conceptualization, Funding acquisition, Methodology, Project administration, Supervision, Writing – review & editing.
