# Supplementary material for: Preoperative prediction of nodal status using clinical data and artificial intelligence derived mammogram features enabling abstention of sentinel lymph node biopsy in breast cancer
Source: Front Oncol. 2024 Jul 10;14:1394448. doi: 10.3389/fonc.2024.1394448 (PMC11266164; doi:10.3389/fonc.2024.1394448)

Supplementary Material

# Supplementary Figures and Tables

## Supplementary Figures


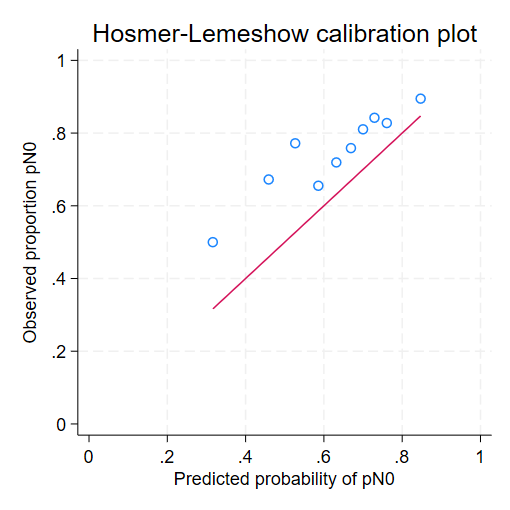


Supplementary Figure 1. Hosmer-Lemeshow calibration plot of the clinical preoperative prediction model in the validation cohort. The prediction probabilities of pN0 were divided into 10 groups of equal size. For each group, the observed fraction pN0 was plotted against the mean predicted probability of pN0. The 45-degree line represents perfect calibration.

## Supplementary Tables

**Supplementary Table 1.** Patient and tumor variables stratified by sentinel lymph node status in the external validation cohort.

|  | **All (n=586)** | **pN0 (n=432)** | **pN+ (n=154)** | ***P*** |
| --- | --- | --- | --- | --- |
| **Age, years (continuous)*^c^** | 66 (29–91) | 66.5 (29–89) | 65 (34–91) | 0.733^a^ |
| **Pathological tumor size, mm (continuous)*^d^** | 15 (1–140) | 13 (4–110) | 20 (1–140) | <0.001^a^ |
| **Mode of tumor detection**^c^** |  |  |  | 0.058^b^ |
| Symptomatic | 240 | 167 (39) | 73 (47) |  |
| Screening | 346 | 265 (61) | 81 (53) |  |
| **Multifocality**^d^** |  |  |  | <0.001^b^ |
| Yes | 149 | 91 (21) | 58 (38) |  |
| No | 429 | 335 (79) | 94 (62) |  |
| Missing | 8 | 6 | 2 |  |
| **Tumor localization**^c^** |  |  |  | 0.001^b^ |
| Central | 8 | 2 (0.5) | 6 (4) |  |
| Upper inner | 74 | 65 (15) | 9 (6) |  |
| Lower inner | 39 | 28 (6.5) | 11 (7) |  |
| Upper outer | 205 | 157 (36) | 48 (31) |  |
| Lower outer | 51 | 37 (9) | 14 (9) |  |
| Overlapping | 207 | 142 (33) | 65 (42) |  |
| Missing | 2 | 1 | 1 |  |
| **Histological type**^c^** |  |  |  | 0.038^b^ |
| NST and lobular | 542 | 393 (91) | 149 (97) |  |
| Other or mixed | 44 | 39 (9) | 5 (3) |  |
| **Histological grade**^c^** |  |  |  | 0.004^b^ |
| I | 141 | 119 (28) | 22 (14) |  |
| II | 339 | 241 (56) | 98 (64) |  |
| III | 103 | 70 (16) | 33 (22) |  |
| Missing | 3 | 2 | 1 |  |
| **Vascular invasion**^d^** |  |  |  | <0.001^b^ |
| Yes | 103 | 52 (12) | 51 (34) |  |
| No | 470 | 373 (88) | 97 (66) |  |
| Missing | 15 | 7 | 8 |  |
| **ER status**^c^** |  |  |  | 0.857^b^ |
| Negative | 36 | 27 (6) | 9 (6) |  |
| Positive | 550 | 405 (94) | 145 (94) |  |
| **PR status**^c^** |  |  |  | 0.310^b^ |
| Negative | 91 | 71 (16) | 20 (13) |  |
| Positive | 495 | 361 (84) | 134 (87) |  |
| **HER2 status**^c^** |  |  |  | 0.082^b^ |
| Negative | 554 | 412 (96) | 142 (92) |  |
| Positive | 30 | 18 (4) | 12 (8) |  |
| Missing | 2 | 2 | 0 |  |
| **Ki67 (continuous)*^c^** | 21 (2.5–93) | 20 (2.5–93) | 26.5 (3–92) | <0.001^a^ |
| **Radiological tumor size, mm (continuous)*^c^** | 16 (3–110) | 14 (3–90) | 20 (6–110) | <0.001^a^ |
| Missing | 55 | 44 | 11 |  |

Abbreviations: Negative sentinel lymph node status (pN0), positive sentinel lymph node status (pN+), no special type (NST), estrogen receptor (ER), progesterone receptor (PR).
*Median (range)
**Number (%)
^a^ Mann–Whitney U test
^b^ Chi-square test
^c^ Preoperatively available
^d^ Postoperatively available

**Supplementary Table 2.** A comparison of pathological and radiological tumor size measurements.

|  |  | **Radiological tumor size** | | |
| --- | --- | --- | --- | --- |
| **Pathological tumor size** |  | ≤10 mm | 11–20 mm | >20 mm |
|  | ≤10 mm | 98 | 25 | 3 |
|  | 11–20 mm | 95 | 146 | 47 |
|  | >20 mm | 12 | 65 | 100 |

**Supplementary Table 3.** Univariable logistic regression of radiologic variables for N0 status.

|  | **N 770** | **OR (95% CI)** | **P** |
| --- | --- | --- | --- |
| **Radiological tumor size, mm (continuous)** | 591 | 0.968 (0.952–0.985) | <0.001 |
| Missing | 179 |  |  |
| **Breast density, % (continuous)** | 748 | 0.998 (0.991–1.005) | 0.605 |
| Missing | 22 |  |  |
| **Dense area, cm^2^ (continuous)** | 748 | 1.004 (0.996–1.004) | 0.859 |
| Missing | 22 |  |  |
| **Highest calc cluster score (continuous)** | 689 | 1.000 (0.996–1.003) | 0.842 |
| Missing | 81 |  |  |
| **Calc cluster (binary)** | 689 |  | 0.976 |
| Absence | 446 | 1 (reference) |  |
| Presence | 243 | 0.995 (0.715–1.385) |  |
| Missing | 81 |  |  |
| **Highest soft tissue lesion score (continuous)** | 689 | 0.997 (0.992 – 1.001) | 0.152 |
| Missing | 81 |  |  |
| **Soft tissue lesion (binary)** | 689 |  | 0.346 |
| Absence | 117 | 1 (reference) |  |
| Presence | 572 | 0.812 (0.528–1.251) |  |
| Missing | 81 |  |  |
| **Mammography malignancy score** | 667 |  | 0.414 |
| 1 | 26 | 1 (reference) |  |
| 2 | 8 | 4.375 (0.466–41.07) | 0.196 |
| 3 | 59 | 1.424 (0.542 – 3.737) | 0.473 |
| 4 | 205 | 1.442 (0.620–3.354) | 0.396 |
| 5 | 369 | 1.109 (0.489–2.513) | 0.804 |
| Missing | 103 |  |  |
| **Ultrasound malignancy score** | 660 |  | 0.107 |
| 1 | 35 | 1 (reference) |  |
| 2 | 8 | 0.750 (0.124–4.546) | 0.754 |
| 3 | 32 | 0.550 (0.180–1.678) | 0.294 |
| 4 | 132 | 0.667 (0.268–1.660) | 0.384 |
| 5 | 453 | 0.428 (0.183–1.002) | 0.050 |
| Missing | 110 |  |  |

Abbreviations: negative sentinel lymph node (N0) status, odds ratio (OR), confidence interval (CI).

**Supplementary Table 4.** AUCs from univariable analyses of radiomic variables.

|  | **AUC (95% CI)** |
| --- | --- |
| Breast density, % (continuous) | 0.53 (0.48–0.57) |
| Dense area, cm2 (continuous) | 0.49 (0.44–0.53) |
| Highest calc cluster score (continuous) | 0.52 (0.48–0.56) |
| Calc cluster (binary) | 0.50 (0.46–0.54) |
| Highest soft tissue lesion score (continuous) | 0.58 (0.53–0.63) |
| Soft tissue lesion (binary) | 0.51 (0.49–0.54) |

Abbreviations: area under the receiver operating characteristic curve (AUC), confidence interval (CI).

**Supplementary Table 5.** Akaike information criterion for the presented models (n=503).

|  | AIC |
| --- | --- |
| Modified postoperative model | 576 |
| Clinical preoperative model | 616 |
| Combined preoperative model | 618 |

Abbreviations: Akaike information criterion (AIC).

**Supplementary Table 6.** Selection percentages when repeating the stepwise variable selection in 1000 bootstrap samples.

|  | **Bootstrap (%)** |
| --- | --- |
| **Radiological tumor size** | 96.5 |
| **Age** | 89.9 |
| **Mode of detection** | 89.7 |
| **Histological type** | 76.2 |
| **Estrogen receptor status** | 97.9 |
| **Tumor localization** | 64.1 |
| **Highest soft tissue lesion score** | 72.5 |
| **Soft tissue lesion (binary)** | 65.6 |
| **Highest calcification score** | 33.3 |
| **Calcification (binary)** | 35.3 |
| **Breast density** | 47.2 |
| **Dense area** | 45.5 |
| **Mammographic malignancy score** |  |
| 1 | (reference) |
| 2 | 25.0 |
| 3 | 49.3 |
| 4 | 49.3 |
| 5 | 48.9 |
| **Ultrasound malignancy score** |  |
| 1 | (reference) |
| 2 | 23.3 |
| 3 | 40.9 |
| 4 | 40.9 |
| 5 | 37.7 |

##
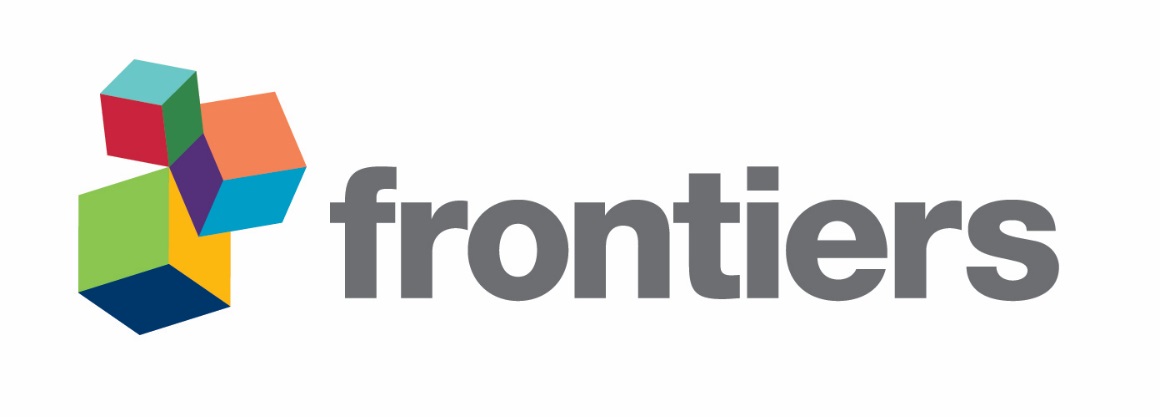

Supplement: Supplementary file 1 [file DataSheet_1.docx]
